# Rhizobium Symbiotic Capacity Shapes Root-Associated Microbiomes in Soybean

**DOI:** 10.3389/fmicb.2021.709012

**Published:** 2021-12-02

**Authors:** Yuanhui Liu, Bin Ma, Wenfeng Chen, Klaus Schlaeppi, Matthias Erb, Erinne Stirling, Lingfei Hu, Entao Wang, Yunzeng Zhang, Kankan Zhao, Zhijiang Lu, Shudi Ye, Jianming Xu

**Affiliations:** ^1^Institute of Soil and Water Resources and Environmental Science, College of Environmental and Resource Sciences, Zhejiang University, Hangzhou, China; ^2^Zhejiang Provincial Key Laboratory of Agricultural Resources and Environment, Zhejiang University, Hangzhou, China; ^3^China National Rice Research Institute, Chinese Academy of Agricultural Sciences, Hangzhou, China; ^4^Hangzhou Global Scientific and Technological Innovation Center, Zhejiang University, Hangzhou, China; ^5^State Key Laboratory of Agrobiotechnology, College of Biological Sciences and Rhizobium Research Center, China Agricultural University, Ministry of Agriculture Key Laboratory of Soil Microbiology, Beijing, China; ^6^Department of Environmental Sciences, University of Basel, Basel, Switzerland; ^7^Institute of Plant Sciences, University of Bern, Bern, Switzerland; ^8^Acid Sulfate Soils Centre, School of Biological Sciences, The University of Adelaide, Adelaide, SA, Australia; ^9^Departamento de Microbiología, Escuela Nacional de Ciencias Biológicas, Instituto Politécnico Nacional, México City, México; ^10^College of Bioscience and Biotechnology, Yangzhou University, Yangzhou, China

**Keywords:** root-associated microbiome, symbiosis, rhizobia, *Glycine max*, root exudation

## Abstract

Root-microbiome interactions are of central importance for plant performance and yield. A distinctive feature of legumes is that they engage in symbiosis with N_2_-fixing rhizobia. If and how the rhizobial symbiotic capacity modulates root-associated microbiomes are still not yet well understood. We determined root-associated microbiomes of soybean inoculated with wild type (WT) or a *noeI* mutant of *Bradyrhizobium diazoefficiens* USDA 110 by amplicon sequencing. UPLC-MS/MS was used to analyze root exudates. The *noeI* gene is responsible for fucose-methylation of Nod factor secreted by USDA 110 WT strain. Soybean roots inoculated with the *noeI* mutant showed a significant decrease in nodulation and root-flavonoid exudation compared to roots inoculated with WT strain. The *noeI* mutant-inoculated roots exhibited strong changes in microbiome assembly in the rhizosphere and rhizoplane, including reduced diversity, changed co-occurrence interactions and a substantial depletion of root microbes. Root exudates and soil physiochemical properties were significantly correlated with microbial community shift in the rhizosphere between different rhizobial treatments. These results illustrate that rhizobial symbiotic capacity dramatically alters root-associated microbiomes, in which root exudation and edaphic patterns play a vital role. This study has important implications for understanding the evolution of plant-microbiome interactions.

## Introduction

The interactions between plants and microbial communities (including archaea, bacteria, fungi, oomycetes, and protists) impact host health, fitness and biogeochemical cycling (Panke-Buisse et al., 2015). The highly dynamic microbial communities that colonize the root-soil interface, so-called rhizosphere, are particularly important in this context (Lundberg et al., 2012). Beneficial effects of root microbiomes include enhanced nutrient acquisition, disease suppression, plant immunity, abiotic stress tolerance, and improved adaptation to environmental variation (van der Heijden et al., 2016). Through the release of root chemicals, plants provide specific niches for root microbiomes which favor the survival and adaptation of specialized inhabitants (Thrall et al., 2007). Apart from plant chemistry, several other factors like soil type, host genotype, developmental stage, nutrient status and rhizosphere-compartmentalization are important determinants of root-associated microbiome assemblages (Cordovez et al., 2019; Brown et al., 2020).

High genetic variation in different members of microbiome communities is frequent in nature (Van Den Broek et al., 2005). The impact of plant genetic variation on root-associated microbiomes is becoming more and more studied (Hu et al., 2018), while much less is known about the importance of microbial genetic variation. So far, microbial genetic variation has mostly been studied in host-pathogen interactions due to the interest in genetic variants enabling pathogens to evade host immunity (Craig and Scherf, 2003). For instance, a mutation of a site-specific recombinase gene in *Pseudomonas fluorescens* WCS365 causes reduced competitive colonization of root tips in tomato (Dekkers et al., 1998). Studies have also uncovered links between microbial genetic variation and the production of secondary metabolites and biocontrol activity of rhizosphere bacteria (Chancey et al., 2002). Evolution is apparent and fast in microorganisms, and root-microbiomes can thus be expected to be genetically dynamic (Lenski, 2017). High-throughput sequencing studies often investigate the taxonomic or functional compositions of root-associated microbiomes, but do not approach the functional consequences of microbial genetic variation on the host plant (Bai et al., 2015). Genetic manipulation of individual strains can help to assess the importance of selected heritable traits in determining the composition and function of microbiomes in the context of root-microbiome interactions.

Over the course of evolution, legumes have developed mutualistic relationships with rhizobia, also called root/stem nodule nitrogen-fixing bacteria. This interaction involves forming nodules in which rhizobia convert atmospheric nitrogen (N_2_) into ammonia (NH_3_) that is used as a nitrogen-resource for the legumes. In turn, legumes supply photosynthates to their bacterial symbionts (Oldroyd et al., 2011; Lindstrom and Mousavi, 2019). Rhizobia-legume symbiosis is diverse (Wang et al., 2012; Lorite et al., 2018). For a given host, functional symbiosis with rhizobia is affected by the competitive ability of rhizobia and by environmental factors such as soil properties (Frey and Blum, 1994). Although it has recently been shown that the rhizosphere microbiome has a crucial regulatory role in shaping rhizobia-soybean symbiosis (Han et al., 2020), little is known about the effect of rhizobia-host symbiosis on root-associated microbiomes.

The legume-rhizobial symbiosis begins with the secretion of flavonoids by the plant’s roots; flavonoid exudates are specifically recognized by certain rhizobia through their NodD receptors (Bolaños-Vásquez and Werner, 1997). This in turn induces the bacterial partner to synthesize and release Nod factors (NFs), which are signal molecules coded by nodulation genes (*nod*, *nol*, and *noe*; Oldroyd et al., 2011). Rhizobial nodulation genes are located on transmissible genetic elements such as symbiotic plasmids or islands and can be transferred horizontally at high frequency within the species (Remigi et al., 2016). On the plant side, lysin motif (LysM) receptor kinases recognize and bind compatible NFs, and then initiate the accommodation of the rhizobia through the nodule-formation process (Oldroyd et al., 2011; Broghammer, 2012). Rhizobial nodulation genes and plant symbiotic signaling genes, including NF receptor genes and downstream common symbiotic signaling pathway (SYM) genes, which are shared with arbuscular mycorrhizal (AM) symbiosis, are necessary to establish the symbiotic relationship and nodule development (Oldroyd et al., 2011; Oldroyd, 2013). Previous studies have shown that genetic variation in plant genes encoding the common SYM receptor in *Lotus japonicas* (Zgadzaj et al., 2016), *Glycine max* (Okubo et al., 2009), *Medicago truncatula* (Offre et al., 2007) and the non-leguminous *Oryza sativa* (Ikeda et al., 2011) drive the establishment of distinctive root-associated microbiomes. In contrast, the impact of microbial genetic variation such as those found in rhizobial nodulation genes on root-associated microbiomes remains unknown. This is particularly relevant in the light of recent findings showing that rhizobia acquired key symbiosis genes multiple times, and that the most recent common ancestor was able to colonize roots of many different plant species (Garrido-Oter et al., 2018), raising the question whether and how evolution of symbiosis affects plant-microbiome interactions.

The common *nod* genes *nodA*, *nodB*, and *nodC* are responsible for synthesizing the core structure of NFs and are necessary for most symbioses, while other nodulation genes encode the specific modifications on the backbone of signaling compounds and have effects on host specificity (Lerouge et al., 1990). The *noeI* gene is responsible for the methylation of the fucose moiety at the reducing end of NFs (Jabbouri et al., 1998). Previous studies have found that *noeI* was not essential for *Sinorhizobium fredii* HH103 and *Sinorhizobium* sp. NGR234 in their nodulation with several host plants (Jabbouri et al., 1998; Madinabeitia et al., 2002). However, a recent study conducted on *Bradyrhizobium diazoefficiens* USDA 110 found that *noeI* has a vital role in maintaining nitrogen fixation efficiency in associating with soybean (Liu et al., 2018). Nodulation phenotypes and host nitrogen status are known to have an impact on the structure of root- and shoot-associated microbiomes in soybean (Ikeda et al., 2010; Klinger et al., 2016), while the effect of rhizobial genetic variation with different nitrogen-fixing efficiency on root-associated microbiomes and underlying mechanisms are largely unknown.

In this study, we investigated the role of genetic variation in the *noeI* gene of *B. diazoefficiens* (strain USDA 110) in regulating the assembly of soybean root-associated microbiota. We sampled five compartments including rhizosphere, rhizoplane, endosphere, nodules, and unplanted soil (Edwards et al., 2015) to determine the direct and plant-mediated effects of the rhizobial wild type and *noeI* gene mutant on the composition and diversity of root-associated bacterial communities. Further, we investigated the potential role of plant flavonoids in triggering these effects. Our results reveal that the wild type of soybean rhizobium with intact *noeI* gene determines the composition of root-associated microbiota through plant and environmental factors mediated effects, such as flavonoid exudation and soil properties. These findings shed light on the mechanisms underlying the relationship between specific root-microbe symbiosis and distinct root-associated microbial communities.

## Materials and Methods

### Soil Field Sampling

Soil samples were collected from a perennially flooded paddy field located in Leshan, Sichuan Province, China (29.2593 N, 103.9403 E). Surface soil was collected at a depth of 0–20cm through a mixed “five point” sampling strategy in a 25m×25m field as described in Ma et al., (2017). The soil was transported immediately to the laboratory on ice and stored at 4°C. Plant residues, roots, and stones were removed, and the soil was drained well enough to pass through a 2mm sieve. This field soil was bulked and subsequently used in greenhouse batch experiments as it contains no native compatible rhizobia that can nodulate with *G. max* variety C08. The basic properties of the soil were: pH 5.3 (soil:water=1:2.5); 1.95% total carbon (TC), 0.16% total nitrogen (TN), 1.01% hydrogen (H), and 0.05% sulphur (S) contents; 23.52mgkg^−1^ NO_3_^−^-N; 28.98mgkg^−1^ NH_4_^+^-N; 16.55 cmolkg^−1^ cation exchange capacity (CEC); 37.58mgkg^−1^ dissolved organic carbon (DOC) and 2.93mgkg^−1^ dissolved organic nitrogen (DON), 0.2497mgkg^−1^ exchangeable sodium (Na^+^), 0.7898mgkg^−1^ exchangeable potassium (K^+^), 4.323mgkg^−1^ exchangeable calcium (Ca^2+^), and 1.72mgkg^−1^ exchangeable magnesium (Mg^2+^). Cultivated soybean (*G. max*) variety C08 was used in this study.

### Greenhouse Experiment and Symbiotic Phenotype Testing

**Figure 1:**
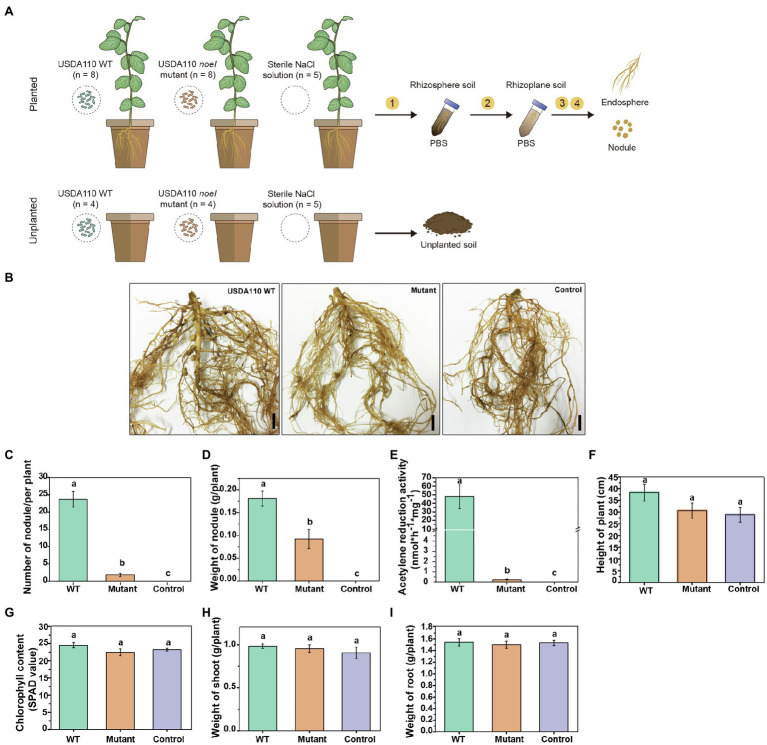
The experimental design and symbiotic phenotypes of soybean inoculated with rhizobia. **(A)** Soybean plants (*Glycine max* C08) were inoculated with *Bradyrhizobium diazoefficiens* USDA 110 WT or *noeI* mutant. Sterile 0.8% NaCl solution was used as control. The rhizosphere soil, rhizoplane soil, endosphere, and nodules were sampled 45days post-inoculation (dpi). In addition, unplanted soil samples treated with the same treatments were collected at 45 dpi. ① Rhizosphere soil samples were collected by vortexed shaking and washing in phosphate-buffered saline (PBS) buffer, ② rhizoplane soil samples were collected from sonicating and washing, ③ endosphere samples were obtained by surface-sterilizing, and ④ nodules were collected from the cleaned roots. **(B)** Images depicting the root system of soybean plants inoculated with the USDA 110 WT or the *noeI* mutant or the control solution (scale bars: 1cm); Scored nodulation phenotypes included **(C)** number of nodules per plant, **(D)** nodule weight, **(E)** nodule nitrogenase activity, **(F)** height of plant, **(G)** leaf chlorophyll content (SPAD), **(H)** dry weight of shoots and, **(I)** dry weight of roots. Means and standard errors are based on 16 scored plants; different letters indicate significant differences among treatments [Least Significant Difference (LSD) test, *p*<0.05].

The greenhouse experiment was of a complete factorial randomized block design (Figure 1A) that consisted of two rhizobial genotype treatments and two planting patterns. The rhizobial genotype treatments included: (1) *B. diazoefficiens* USDA 110 wild type (WT), isolated from soybean (Delamuta et al., 2013); and (2) *B. diazoefficiens* USDA 110 *noeI* mutant, obtained in our previous study (Liu et al., 2018). The two planting patterns were (1) planted with cultivated soybean (variety C08) and (2) intact soil without plants (unplanted). Planted and unplanted soils inoculated with sterile 0.8% NaCl (*w*/*v*) solution were included as negative control treatments. Each planted treatment had two plants per pot; each pot was a replicate. The planted pots inoculated with WT and mutant USDA 110 contained eight replicates each (Figure 1A); unplanted pots for these treatments contained four replicates each. The negative controls had five replicates regardless of planting status.

Soybean seeds were selected for fullness and uniformity before being surface-sterilized in 95% ethanol for 30s and then further sterilized with 2.5% (*v*/*v*) sodium hypochlorite (NaClO) solution for 3–5min, after which they were rinsed seven times with sterilized deionized water. The surface-sterilized seeds were germinated on 0.8% water-agar (*w*/*v*) plates in the dark at 28°C for 36–48h. Uniform germinated seedlings were selected and transferred into pots (10 by 12cm height by diameter) containing 500g of soil. Each treatment was inoculated with 1ml of rhizobial culture with optical density at 600nm of 0.2, which was centrifuged, washed and resuspended in 0.8% NaCl solution as described in our previous study (Liu et al., 2018). Plants were grown in the greenhouse (day/night cycle 16/8h, 25/16°C and a relative humidity of 60%) and were harvested 45days post-inoculation (dpi), which was 5days later than usual as the *noeI* mutant may have delayed nodulation. Several symbiotic phenotypes were recorded for plants inoculated with the wild type and the mutant. Leaf chlorophyll concentrations were determined using a SPAD-502 meter (Konica Minolta, Osaka, Japan; Ling et al., 2011). Plant height, weight of fresh nodules and the number of nodules were measured after sampling and shoot and root weights were determined after being dried at 65°C for 5days. Nodule nitrogenase activity was measured using the acetylene reduction method (Buendiaclaveria et al., 1989).

### Sampling of Unplanted Soil, Rhizosphere, Rhizoplane, Endosphere, and Nodule

The method for sampling unplanted soil, rhizosphere, rhizoplane, endosphere, and nodules followed the protocol described previously (Edwards et al., 2015) with modifications. Briefly, the plants were removed from each pot and the loosely attached soil on the roots was removed with gentle shaking, leaving the root-adhering soil layer (approximately 1mm of soil). The soil collection steps were performed on ice. Firstly, the roots were placed in a sterile 50ml sterile centrifuge tube containing 30ml of sterile pre-cooled phosphate-buffered saline (PBS) buffer (with pH 7.3–7.5) and vortexed for 15s, the turbid solution was filtered through a 100μm aseptic nylon mesh strainer into a new 50ml tube to remove root fragments and large sediments, followed by centrifuging for 5min at 12,000×*g* at 4°C. The supernatant was discarded, and the soil washed from the roots was defined as rhizosphere soil, which was then frozen with liquid nitrogen and stored at −80°C. For rhizoplane samples, the washed roots were transferred to a sterile centrifuge tube with 30ml PBS and sonicated for 30s at 50–60Hz twice. The roots were then removed, and the rhizoplane samples was collected by centrifugation at 12,000×*g* for 5min at 4°C and stored at −80°C until DNA extraction. The washed roots were cleaned and sonicated again as described before to ensure that all microbes were removed from the root surface. Two more sonication procedures using clean PBS solution were performed, and the sonicated roots were surface-sterilized in 70% (*v*/*v*) ethanol for 2min and then in 2.5% (*w*/*v*) NaClO solution for 5min, followed by washing with PBS solution seven times. The root nodules were collected by separating them from roots using sterile blades. The roots were defined as endosphere samples and stored at −80°C alongside the nodules. Unplanted soil samples were collected from unplanted pots approximately 2cm below the soil surface and stored at −80°C until DNA extraction.

### DNA Extraction, 16S rRNA Gene Sequencing and Analysis

Metagenomic DNA of each sample was extracted using the FastDNA Spin Kit for Soil (MP Biomedicals, LLC., Solon, OH, United States) following the manufacturer’s protocol. DNA concentration and purity were evaluated photometrically using a NanoDrop ND-1000 UV-Vis spectrophotometer (NanoDrop Technologies, Wilmington, DE, United States). The extracted DNA was stored at −80°C until further analysis. Primers 515F (5′-GTGCCAGCMGCCGCGGTAA-3′) and 806R (5′-GGACTACHVGGGTWTCTAAT-3′) were used to amplify the variable V4 region of the bacterial 16S rRNA gene. PCR conditions were as follows: 94°C, 5min, followed by 30cycles of amplification (94°C, 30s; 52°C, 30s; 72°C, 30s), and finally 72°C, 10min. Sequencing libraries were generated using NEBNext Ultra DNA Library Prep Kit for Illumina (New England Biolabs, MA, United States) following the manufacturer’s recommendations and index codes were added. The library quality was assessed on a Qubit 2.0 Fluorometer (Thermo Fisher Scientific, MA, United States) and an Agilent Bioanalyzer 2100 system (Agilent Technologies, Waldbronn, Germany). Finally, the library was sequenced on an Illumina_Hiseq2500 platform and paired-end reads of length 250bp were generated (Guangdong Magigene Biotechnology Co., Ltd. Guangzhou, China). The resulting paired sequence reads were then merged, trimmed, filtered, aligned, and clustered to define the operational taxonomic unit (OTU) using USEARCH v.11.06 (Edgar, 2013). Briefly, sequences with ≥97% similarity were assigned to the same OTU by the UPARSE-OTU algorithm in USEARCH; and chimera detection was performed with VSEARCH 2.11 (Rognes et al., 2016). Putative chimeric sequences and singletons were discarded; the taxonomical alignment was determined using the RDP (Ribosomal Database Project) classifier.

### Root Exudate Collection and UPLC-MS/MS Analysis

Full and uniform soybean seeds were surface sterilized and germinated as described above. To enhance root growth, germinated seedlings were transferred to sterile pots containing sterile vermiculite and grown in the greenhouse for 7days under the same conditions as described above. At harvest, the soybean plants were pulled from their pots and washed to remove the vermiculite, then four plants were transferred to a 9-well sponge lattice placed in a glass jar (12.6cm in height and 8.5cm in diameter) containing 100ml 25% (*v*/*v*) of sterile modified Rigaud-Puppo solution supplemented with the same amount of inorganic nitrogen as that in the soil (Rigaud and Puppo, 1975). The plant roots grew through the holes of the lattice into the nutrient solution. These hydroponics systems were inoculated with 4ml of USDA 110 WT and *noeI* mutant cultures as described above with 4ml 0.8% NaCl added to the control samples. To provide an aerobic environment for rhizobia, oxygen was pumped into the nutrient solution; each treatment contained three replicate hydroponics systems. The systems were incubated for 7days in a climate-controlled growth chamber (day/night cycle 14/10h, 28/16°C and relative humidity of 60%). To check the sterility of the hydroponics systems, an aliquot of 500μl from each system was spread and cultured on tryptone-yeast (TY) medium plates. Soybean root exudates were collected by centrifugation at 10,000rpm for 20min (5°C), filtered using a 0.25-μm cellulose nitrate filter and then stored at −20°C until further analysis.

Eleven standard flavonoids (supplied by J&K Scientific Ltd.) were determined during the experiment: naringenin, hesperetin, genistein, daidzein, 7,4′-dihydroxyflavone, apigenin, chrysin, luteolin, isoliquiritigenin, morin and coumestrol; deuterated genistein was used as the internal standard. The calibration curve was prepared by the serial dilution of a mixture of 11 standards by methanol with concentrations as follows: 50, 25, 10, 5, 1, 0.5, 0.1μgL^−1^. The internal standard was also added to all samples to achieve a final concentration of 10μgL^−1^. The calibration curve was obtained by plotting the peak area ratio (*y*) of the standard to the internal standard vs. the ratio of their concentrations (*x*). The curve was fitted to a linear function with a weight of 1/*nx* (*R*^2^>0.99), with “*n*” being the calibration level. The concentrations of the compounds in the sample were determined by their peak area ratio with the internal standard and were determined using the calibration curve. All standards and samples were filtered through a PTFE syringe filter (0.22μm) and stored at −80°C until further analysis.

The internal standard was added to each hydroponic culture (100ml) to give a concentration of 10μgL^−1^ after which the solution was passed through a Resprep C18 solid-phase extraction cartridge [Sep-Pak Vac 6cc (500mg), Waters, United States]. Flavonoids were eluted by 10ml methanol and then freeze-dried with liquid nitrogen. For quantification, samples were resuspended in 1ml of 50% (*v*/*v*) methanol solution and 10μl aliquots were injected into a Waters ACQUITY I-class UPLC coupled with Xevo TQ-XS Triple Quadrupole Mass Spectrometer in the electrospray ionization negative mode (Waters, United States). Liquid chromatography was performed on a 100mm×2.1mm BEH C_18_ column with a particle size of 1.7μm. The mobile phase consisted of solvent A (water) and solvent B (100% acetonitrile) and the flow rate was 0.3ml/min. The optimized linear gradient system was as follows: 0–1min, 5% B; 1–10min, 35% B; 10–12min, 95% B; 12–15.5min, re-equilibrium to 5% B. The parameters of the mass spectrometer were as follows: capillary voltage 2.5kV, cone voltage 80V, desolvation temperature 600°C, desolvation gas flow 1,100Lh^−1^, cone gas flow 250Lh^−1^, nebulizer gas flow 7bar, and collision gas flow 0.15mlmin^−1^ of argon. A multiple reaction monitoring (MRM) mode was employed for quantitative analysis. Mass spectral parameters were optimized for each analyte and are shown in Supplementary Table S1.

### Impacts of the Flavonoid Mixture on Soil Microbiome

To determine the effect of flavonoids on soil microbial community structures, solutions were prepared containing a mixture of the 11 flavonoid standards according to the quantitative analysis of flavonoids secreted by soybean. The final concentration of daidzein was 1μgg^−1^ soil, and the other 10 flavonoids were added following their ratios to daidzein. Stock solutions were generated using serial dilution, with the flavonoids initially dissolved in methanol and thereafter diluted using sterilized water. From the soil described above, 100g were placed into pots and pre-incubated under the greenhouse conditions described above for 1week to activate the soil microbiomes. One milliliter of the mixture solution was added into each pot twice a week for 4weeks. The control treatment had the same volume of sterile water added; each treatment consisted of three replicates. All pots were watered twice a week during the incubation period. The soil samples were collected after incubation, with DNA extracted and the community 16S rRNA genes sequenced and analyzed as described above.

### Physicochemical Characterization of Soil

The soil physicochemical characteristics of each treatment were measured following the methods described by Bao (2000). Soil pH was measured using a suspension of soil and deionized water at a ratio of 1:2.5 (*w*/*v*). Soil total C, N, H, and S contents were determined separately using an elemental analyzer (Flash EA 1112, Thermo Finnigan). DOC and DON were measured using a TOC analyzer (Multi N/C 3100, Analytik Jena AG). Soil exchangeable Na^+^, K^+^, Ca^2+^, and Mg^2+^ were extracted with 1M ammonium acetate from the bulk soil in unplanted treatments and from the rhizosphere soil in the planted treatments. Extracts were measured by atomic absorption spectrophotometry (NovAA300, Analytik Jena AG). NO_3_^−^-N, NH_4_^+^-N, and CEC were measured in a continuous colorimetric flow system (Skalar SAN++ System, Netherlands).

### Statistical Analysis

The resulting OTU table was normalized by the negative binomial model using the package *phyloseq* in R (version 3.6.0). Weighted UniFrac distances were calculated from the normalized OTU tables using the R package *vegan*, principal coordinate analyses (PCoA) utilizing the weighted UniFrac distances to assess the differences in microbial communities between treatments. To measure the *β*-diversity significance, permutational multivariate analyses of variance (PERMANOVA) was conducted using the function *adonis* in *vegan*. Shannon, Chao1 and Fisher indices and the number of observed species were calculated using the function *diversity* in R package *vegan*. Kruskal–Wallis tests followed by Dunn’s multiple-comparison test were performed to assess differences between treatments. Statistical analysis of taxonomic and functional profiles (STAMP) was applied to identify different species associated with rhizobial treatments. Weighted UniFrac distance-based redundancy analysis (db-RDA) and variation partitioning analysis (VPA) were performed using the functions *capscale* and *varpart* in the package *vegan*. To determine OTU enrichment in each treatment, a generalized linear model (GLM) approach using *edgeR* was conducted. Microbial co-occurrence networks were constructed based on Spearman correlations among 300 dominant OTUs for bulk soil+WT samples and bulk soil+*noeI* mutant samples. The nodes in this network represent OTUs and links indicate potential microbial interactions. We adjusted all values *p* of the correlation matrix using the Benjamini and Hochberg FDR controlling procedure. The indirect correlation dependencies were distinguished using the network deconvolution method (Feizi et al., 2013). The subnetworks for various compartments were induced based on OTU-presentation in corresponding samples. The cutoff for correlation value was determined through random matrix theory (RMT)-based methods (Luo et al., 2006). Network properties were calculated with the *igraph* package in R and visualized in Gephi 0.8.2. Fisher’s Least Significant Difference (LSD) test (*p*<0.05) and Duncan multiple-comparison test (*p*<0.05) using R package *agricolae* were employed to analyze the difference of soybean symbiotic phenotypes and relative abundance of bacterial taxa, respectively. All figures in this study were generated using *ggplot2* in R and OriginPro 2017.

## Results

### A Mutation in *noeI* of *Bradyrhizobium diazoefficiens* Suppresses Soybean Nodule Formation

Nodulation genes are essential for the establishment of symbiosis between legumes and rhizobia. To confirm the role of *noeI* in nodulation, we inoculated soybean roots with the WT and *noeI* mutant of strain *B. diazoefficiens* USDA 110 and then screened the root nodule formation after 45 dpi (Figure 1A). Inoculation with WT strain resulted in the formation of >20 nodules per plant, with a total average weight of >17g per plant and a nitrogenase activity of >45nmolh^−1^ mg^−1^ of nodule (Figures 1B–E). Since there are differences in the current culture conditions (soil) compared to previous culture conditions (vermiculite), the mutation of rhizobial *noeI* gene not only impaired the nodule nitrogenase activity but also nodulation capacity of USDA 110 with soybean (Figures 1B–E). The number of nodules was reduced to <2 per plant, and the nitrogenase activity dropped to <0.5nmolh^−1^ mg^−1^ in soybean inoculated with noeI mutant. No nodule formed in plants grown in soils treated with the sterile control solution, and no nitrogenase activity was detected, meaning native nodule-forming rhizobia were absent in the experimental soil (Figure 1B). As plants were well-fertilized, plant height, leaf chlorophyll content and shoot and root dry weights did not differ among treatments (Figures 1F–I), thus allowing us to assess the impact of *noeI* gene-dependent symbiosis on microbial communities independently of plant performance.

### Compartment-Specific Modulation of Microbial Communities by *noeI*

**Figure 2:**
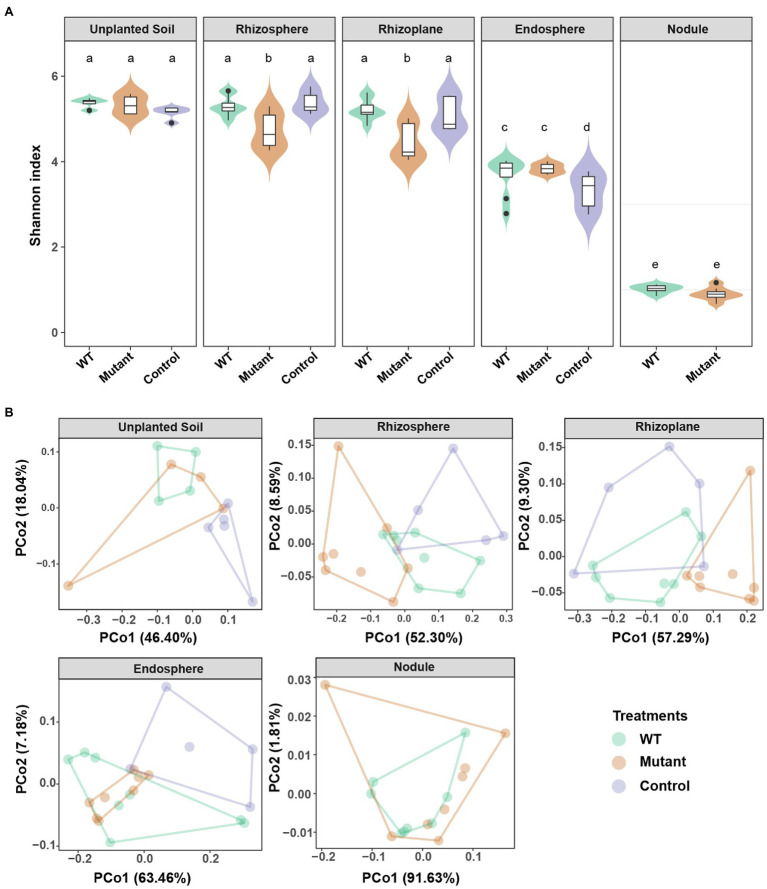
Compartment-specific modulation of microbial communities by rhizobia differing in *noeI* gene. **(A)** α-diversity (Shannon index) among different rhizobial treatments in the unplanted soil, rhizosphere, rhizoplane, endosphere, and nodule compartments. Treatments are wild-type USDA 110 (WT), *noeI* mutant (Mutant), and not inoculated with rhizobia (Control). Different letters indicate significant differences among treatments (Dunn’s multiple-comparison test; *p*<0.05). **(B)** β-diversity principal coordinate analysis (PCoA; weighted UniFrac distances) of unplanted soil, rhizosphere, rhizoplane, endosphere, and nodule communities of soybean inoculated with wild type and mutant rhizobia, and of the control.

To determine whether the *noeI* gene mutation of *B. diazoefficiens* altered the unplanted soil and/or soybean root-associated microbiomes, DNA was extracted from all rhizo-compartments and bacterial community profiles were determined using amplicon sequencing of the 16S rRNA gene. After quality filtering and chimera removal, 6,302,405 sequences (mean, 68,504 per sample) were obtained from 92 samples and 5,667 microbial OTUs were identified at 97% sequence similarity. Alpha diversity was measured using Shannon, Chao1 and Fisher indices as well as with the number of observed OTUs (richness). Alpha diversity was the highest in soil, rhizosphere and rhizoplane; intermediate in the root endosphere; and the lowest in root nodules (Figure 2A, Supplementary Figure S1). In the rhizosphere and rhizoplane compartments, α-diversity was similar following WT- and control-inoculation, but significantly lower following inoculation with the *noeI*-mutant (*p*<0.05; Figure 2A, Supplementary Figure S1). In the endosphere, α-diversity was higher in WT- and *noeI* mutant-inoculated samples than control samples (Figure 2A, Supplementary Figure S1). No differences between treatments were found in unplanted soil and nodules (Figure 2A, Supplementary Figure S1). To exclude this community shifts caused by the relative abundance of the respective inoculants, we determined the alpha diversity of microbial community among the different rhizobial treatments in the absence of the 16S rRNA sequences of *B. diazoefficiens* USDA 110 (OTU_77). The results were similar to those observed in the presence of OTU_77 (Supplementary Figure S2). PCoA and PERMANOVA were performed using weighted UniFrac distances. Samples were separated by both the compartments (39.04% of variation explained, *p*<0.001, Supplementary Figure S1), and inoculation treatments (12.91%, *p*<0.001, Figure 2B, Supplementary Table S2). Furthermore, a significant interaction between compartments and treatments was detected (18.34%, *p*<0.001; Supplementary Table S2). Treatment effects were detected in samples from the rhizosphere (39.88%, *p*<0.001), rhizoplane (36.90%, *p*<0.05), and endosphere (25.63%, *p*<0.03; Figure 2B). Microbial community composition in the rhizosphere and rhizoplane were comparable in WT-inoculated and control roots, but differed significantly in *noeI* mutant-inoculated roots. In the endosphere and unplanted soil, WT and *noeI* mutant-inoculated samples showed similar profiles, but were different from control samples. In the nodules, WT and *noeI* mutant-inoculated showed similar microbial profiles. These results were confirmed by PERMANOVAs (Supplementary Table S2). The beta diversity of microbial community change among different rhizobial treatments was not caused by rhizobial inoculation itself, but by symbiosis with soybean (Supplementary Figure S3). Taxonomy analysis revealed differences in the relative abundance of taxa at class level between WT and mutant treatments in the rhizosphere and rhizoplane; most bacterial classes were less abundant in the samples inoculated with the *noeI* mutant when compared to those inoculated with the WT strain (Supplementary Figure S4). This relationship was not observed in unplanted soil (Supplementary Figure S4). Ktedonobacteria, Planctomycetia, Caldilineae, and Sphingobacteria classes differed significantly between WT and mutant treatments in the rhizosphere (*p*<0.05; Supplementary Figure S4). The relative abundance of the predominant bacterial classes was significantly different between unplanted soil and endosphere compartments (*p*<0.05), but the differences between WT and mutant treatments were not distinct (Supplementary Figure S4). As expected, a pattern of reduced microbial complexity and significantly different relative abundance was found in nodules compared to those of unplanted soil (*p*<0.05; Supplementary Figure S4). Taxonomic assignments at the family level using relative abundance revealed that the nodules in both treatments were dominated by bacteria belonging to the families Bradyrhizobiaceae and Nannocystaceae (Supplementary Figure S5). Furthermore, the 16S rRNA sequences of *B. diazoefficiens* USDA 110 mapped to the most abundant OTU (OTU_77) and accounted for 67.85 and 69.70% of the nodule profiles inoculated with WT and the mutant strain, respectively (Supplementary Figure S5). These results show clearly that the mutation in *noeI* gene of USDA 110 has compartment-specific effects on microbial communities.

### *noeI* Affects Niche Differentiation in Different Rhizo-Compartments

**Figure 3:**
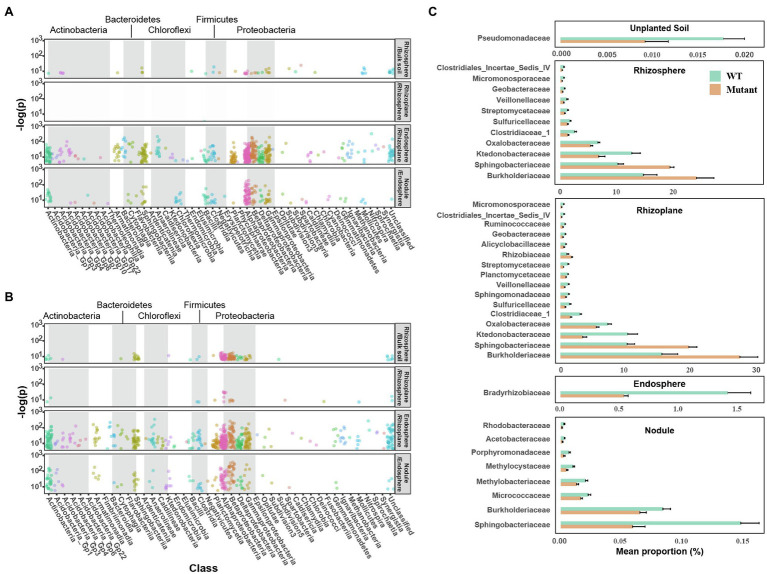
Difference of rhizobial *noeI* affects niche differentiation in the rhizosphere. Bubble plots showing niche differentiation of rhizo-compartments in soybean roots inoculated with wild type (WT) USDA 110 **(A)** and *noeI* mutant rhizobia **(B)**. Compartment X/compartment Y (e.g., Rhizosphere/Bulk soil) represents the significantly enriched OTUs in compartment X relative to compartment Y (*p*<0.05), bubble color indicates operational taxonomic unit (OTU) taxonomic affiliation (class), and grey boxes indicate the OTU taxonomic affiliation (phylum). **(C)** Differences in taxonomic abundance between the WT and *noeI* mutant treatments in unplanted soil, rhizosphere, rhizoplane, endosphere, and nodule samples at the family level (STAMP; Welch’s *t*-test; *p*<0.05).

Enrichment analysis of OTUs using a GLM confirmed differentiation of microbial communities across the rhizo-compartments. Compared to bulk soil, 49 bacterial OTUs mainly assigned to Proteobacteria (Alpha-, Delta-, Beta-, and Gamma-proteobacteria), and the OTUs of Firmicutes (Bacilli, Clostridia) were significantly enriched in the rhizosphere of soybean inoculated with WT strain (Figure 3A). Only one OTU (*Bacillus*) was differentially enriched in the rhizoplane compared to the rhizosphere in the WT treatment. A total of 537 OTUs belonging to the phyla Proteobacteria, Bacteroidetes, Planctomycetes, Actinobacteria, Firmicutes, and Chloroflexi were also enriched in the endosphere compared to the rhizoplane. Overall, 171 OTUs, mainly consisting of Proteobacteria, Firmicutes, Bacteroidetes, Actinobacteria, and Chloroflexi were enriched in nodules compared to the endosphere (Figure 3A). The pattern of microbial community differentiation across the compartments in *noeI* mutant-inoculated samples differed in the rhizosphere and rhizoplane (Figure 3B). Specifically, 148 OTUs were enriched in the rhizosphere relative to the bulk soil, which mainly belonged to Proteobacteria, Bacteroidetes, and Actinobacteria. Compared to WT samples, the rhizoplane enriched a larger proportion of OTUs relative to the rhizosphere (27 vs. 1), which were mainly identified as members of Alphaproteobacteria, Betaproteobacteria, and Clostridia. The microbial community differentiation between endosphere and nodule in mutant-inoculated samples was similar to WT-inoculated samples (Figure 3B).

The STAMP method was performed to identify differences in taxonomic abundances between the WT and mutant treatments at the family level. Only Pseudomonadaceae were significantly enriched in the unplanted soil inoculated with the WT strain compared to that inoculated with the *noeI* mutant (Figure 3C). A total of 11 families and 16 families were found to be significantly (*p*<0.05) different between the inoculated soybean plants in the rhizosphere and the rhizoplane, respectively (Figure 3C). Almost all of the differential families were enriched in samples inoculated with the WT strain, such as Micromonosporaceae, Streptomycetaceae, Clostridiaceae, Geobacteraceae, and Sphingomonadaceae. Strikingly, only Bradyrhizobiaceae in the endosphere samples showed a difference (significantly enriched) with the WT stain treatment (Figure 3C). Finally, eight bacterial families were enriched in nodules of plants inoculated with the WT strain; large differences were observed in Burkholderiaceae and Sphingobacteriaceae (Figure 3C).

### *noeI* Shapes Microbial Co-occurrence Networks

**Figure 4:**
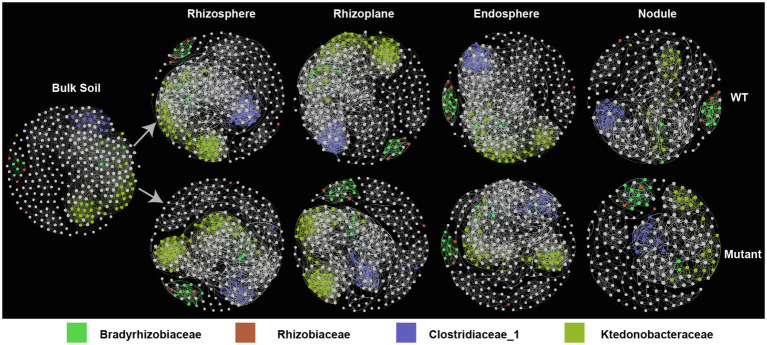
Co-occurrence of networks of rhizo-compartments in samples inoculated with rhizobia. The networks were constructed based on Spearman correlation analysis of taxonomic profiles; *p*<0.05. Node size is proportional to degree; color indicates taxa (family); wild type (WT); *noeI* mutant (Mutant).

To determine whether the *noeI* mutation of *B. diazoefficience* affects co-abundance patterns between bacterial taxa across different rhizo-compartments, we first generated two full networks using bulk soil plus WT or bulk soil plus mutant samples using relative abundances of the 300 most abundant OTUs. We then constructed sub-networks for each rhizo-compartment from the corresponding full networks. In networks of WT- and *noeI* mutant-inoculated samples, the number of nodes and correlations in the sub-networks decreased from rhizoplane to nodule, with no differences between rhizosphere and rhizoplane (Figure 4, Supplementary Table S3). Three dominant clusters were identified in all sub-networks. The first cluster consisted of Bradyrhizobiaceae and Rhizobiaceae families; the second cluster contained taxa from Ktedonobacteraceae; and the third cluster contained families from Clostridiaceae_1. This third cluster exhibited a greater number of connections in the sub-network from samples inoculated with the WT than that in the *noeI* mutant treatment (Figure 4). The topological features of sub-networks differed in both rhizo-compartments and treatments (Supplementary Table S3). Specifically, the average degree of sub-networks decreased from bulk soil to nodule; the sub-network in bulk soil had the lowest modularity, diameter and number of clusters. The average path length, betweenness centrality and modularity of sub-network were highest in the nodule compared to the other rhizo-compartments (Supplementary Table S3). In the rhizobial treatments, the average degree, connectivity and number of clusters of sub-networks were higher in samples inoculated with the WT rhizobium than those inoculated with the mutant, whereas the average path length and diameter were lower in the WT treatment (Supplementary Table S3). Thus, the *B. diazoefficience* with *noeI* mutation alters network topology features of microbial co-occurrence in different rhizo-compartments.

### Role of Flavonoid Exudates and Edaphic Factors in *noeI*-Dependent Effects

**Figure 5:**
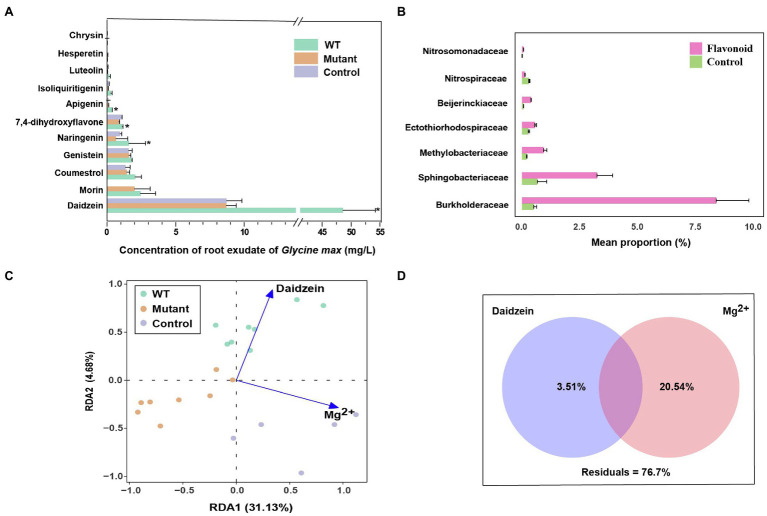
Role of flavonoid exudates in *noeI*-dependent effects. **(A)** Flavonoid concentration in exudates collected from soybean roots inoculated with wild type (WT) and *noeI* mutant (Mutant) rhizobia, and from uninoculated soil (Control); ^*^ indicates significant differences among treatments (LSD test, *p*<0.05). **(B)** Taxonomic abundance differences between soil supplemented with flavonoids and control (STAMP; Welch’s *t*-test, *p*<0.05). **(C)** Redundancy analysis (RDA) of rhizosphere microbial community distribution and environmental factors, soil exchangeable magnesium (Mg^2+^). **(D)** The effects of dominant environmental factors on the structure of microbial communities in rhizosphere [variation partitioning analysis (VPA) independent variance; value <0 not shown].

To determine the potential role of flavonoid exudation in microbial community shift mediated by *noeI*, we collected exudates from soybean plants following inoculation with WT and *noeI*-mutant of strain USDA 110 and analyzed them by UPLC-MS/MS. Eleven flavonoids were identified and quantified in soybean root exudates (Figure 5A). Compared to control roots, WT-inoculation increased the exudation of the six most abundant flavonoids, including a fivefold increase in daidzein (*p*<0.05; Figure 5A). These increases were absent in exudates of plants inoculated with the *noeI*-mutant, whose flavonoid exudation profiles were similar to those of control roots (Figure 5A). Further, we performed an incubation experiment using the same soil and supplemented it with a mixture of flavonoids, which contained 1μgg^−1^ daidzein and the other 10 flavonoids twice a week. The observation on changes in bacterial community structure using amplicon sequencing after 4weeks of incubation revealed that the alpha diversity was lower in the soil treated with flavonoids than that in the control treatment (Supplementary Figure S6A). PCoA using weighted Unifrac distances indicated a distinct separation compared between soil treated with flavonoids and treated with water (control; Supplementary Figure S6B). This was confirmed by PERMANOVA with 46.60% of variance (*p*<0.001). STAMP analysis revealed that the families of Burkholderiaceae, Methylobacteriaceae and Sphingobacteriaceae were significantly enriched in the soils treated with flavonoids compared to the control (Figure 5B). We next explored the relationships between environmental factors and rhizosphere bacterial community composition by weighted UniFrac distance-based redundancy analysis (db-RDA; Figure 5C). The environmental factors included flavonoids and several soil chemical factors, such as TC, TN, DOC and DON, pH, CEC, the majority of which were highly correlated with each other (Supplementary Figure S6C). The weak self-correlation of daidzein and soil exchangeable Mg^2+^ were used as representatives to analyze the relationship between environmental factors and microbial communities’ shift. Daidzein represents all tested environmental factors except CEC, chrysin, and hesperetin, while soil exchangeable Mg^2+^ represents the rest based on correlation analysis (Supplementary Figure S6C). The environmental factors represented by daidzein were identified to explain the rhizosphere microbial communities shift between the WT and mutant treatments. By contrast, soil exchangeable Mg^2+^, CEC, chrysin, and hesperetin explained differences in microbiomes between rhizospheres of control and inoculation treatments (*p*<0.001; Figure 5C). To further assess the contribution of soil exchangeable Mg^2+^ and daidzein to the diversity of microbial community in rhizospheres, variance partitioning analysis was applied; this metric indicated that soil exchangeable Mg^2+^ and daidzein explained 20.54 and 3.51% of microbial community variation, respectively (Figure 5D). These results indicate that most flavonoid exudation and soil physiochemical properties may be related to the differences in microbial community composition that are triggered by intact *noeI* gene-containing *B. diazoefficience* WT strain.

## Discussion

In this study, a genetic variation (a single *noeI* gene mutation) is used to demonstrate that the symbiotic capacity of rhizobial strain (*B. diazoefficiens*) with soybean has major effects on root-associated microbiomes through plant-mediated interactions. Here, we discuss these findings in the context of root-microbiota interactions.

### Supressed Symbiotic Efficiency With Soybean Caused by *noeI* Mutant

The nodulation tests in the present study show that the mutation of *noeI* in rhizobia significantly reduced nodule nitrogenase activity, as reported previously (Liu et al., 2018), and reduced nodulation efficiency of *B. diazoefficiens* with soybean as the soybean plants inoculated with mutant *B. diazoefficiens* had fewer but bigger nodules compared to plants inoculated with WT strain (Figure 1). These results imply that the *noeI* gene is responsible for symbiosis efficiency, but not for host specificity, and also confirmed that the host legume can make conditional sanctions, supplying resources to the rhizobial strains with poor ability of nitrogen fixation when the better symbiotic partners were absent (Westhoek et al., 2021). It is well known that the rhizobia-legume interaction is highly specific and widely diverse (Wang et al., 2012; Lorite et al., 2018). The successful mutualistic association depends not only on the compatibility but also the ability of rhizobia to adapt various environmental factors, such as soil pH and nitrogen content (Hungria and Vargas, 2000; Yang et al., 2018). Soybean is considered a promiscuous species for its ability to nodulate with several bacterial species and genera; *Sinorhizobium* and *Bradyrhizobium* are the two main groups (Yang et al., 2018). It has been reported that *Bradyrhizobium* strains, such as *B. diazoefficiens* USDA 110, are predominant in nodules of soybean cultivated in acidic soil, while *Sinorhizobium* strains are predominant in nodules of soybean cultivated in alkaline soil (Tian et al., 2012). Khan and Khan (1981) found that the number and weight of nodules significantly decrease with increasing levels of NO_3_^−^ in soil. In this study, we determined if and how the symbiotic capacity of *B. diazoefficiens* USDA 110 with soybean modulates root-associated microbiomes. To eliminate the effects of indigenous rhizobia in soil, we collected six soil samples from different provinces in China and performed the nodulation test using cultivated soybean variety (*G. max* C08). The soil from Leshan was chosen as it has no native and compatible rhizobia symbiosis with variety C08 and the pH (5.3) is conducive to the growth of USDA 110. Further, the nitrogen content in the soil is not high to significantly inhibit nodulation. The host plant phenotypes, including plant height, leaf chlorophyll content and weight of shoot were higher in soybean inoculated with the WT strain than those without inoculation (control), although they did not reach significant levels. These responses indicate that the rhizobia we inoculated indeed contributed to the growth of soybean. The absence of a significant difference in plant phenotypes might be explained by the N-content in experiment soil may fit the requirement of soybean in our test period. The difference of plant phenotypes between inoculation and control will be obvious when the soil nitrogen content cannot fit the growth of soybean without inoculation (control).

### Compartment-Specific Microbial Community Shifts Caused by *noeI* Mutant

Our bacterial community sequencing approach evidenced a clear differentiation of bacterial community structure between unplanted soil, rhizosphere, rhizoplane, endosphere, and nodule compartments, with a gradient of decreasing bacterial α-diversity from rhizosphere to endosphere and to nodules. This observation is consistent with previous studies on microbiomes associated with various plants, including of *M. truncatula* (Brown et al., 2020), *L. japonicus* (Zgadzaj et al., 2016), soybean (Mendes et al., 2014), pea (Turner et al., 2013), peanut (Chen et al., 2014) and rice (Edwards et al., 2015). Interestingly, disrupting symbiosis between *B. diazoefficiens* and soybean significantly reduced bacterial diversity in the rhizosphere and rhizoplane. This result is in line with a recent study on plant SYM mutants that documented a reduction of fungal diversity (Thiergart et al., 2019). In contrast to the root-associated compartments, there was no effect of the *noeI* mutation on the diversity and composition of bacterial communities in the absence of soybean plants. This shows that the effect of *noeI* on bacterial communities is plant-mediated. As a gene involved in nodulation, *noeI* is only expressed under induction of flavonoids secreted from the host plant (Jabbouri et al., 1998). Thus, the impact of the *noeI* mutation is indeed expected to be restricted to the interaction between the plant and *B. diazoefficiens*. Previous studies using SYM mutants in *M. truncatula* (Offre et al., 2007), *L. japonicus* (Zgadzaj et al., 2016), and soybean (Okubo et al., 2009), demonstrated significant effects of these mutations on root-associated microbial community assemblages. Even in non-leguminous plants such as *O. sativa*, a mutated SYM pathway gene (*CCaMK*) has been found to structure distinctive root-associated microbiomes, as reflected by enrichment in Rhizobiales and Sphingomonadales (Ikeda et al., 2011), thus complicating the interpretation of these results in the context of legume symbiosis. Our work strengthens the notion that the successful establishment of legume symbiosis has substantial knock-on effects on native legume root-associated microbiota. It is likely that these changes will impact plant performance and soil legacy effects, thus influencing plant productivity in nature and agriculture beyond the primary effect of the symbiosis. Understanding these consequences is an exciting prospect of this work.

In contrast to the WT enriched OTUs, Bradyrhizobiaceae was depleted in soybean root endosphere inoculated with the *noeI* mutant, even though there was no significant difference in *Bradyrhizobium* abundance between WT and mutant-induced nodules. This is consistent with the previous report that a parallel rather than consecutive selection of bacterial taxa from the rhizosphere assemblage might occur for enrichment in the endosphere and nodule (Zgadzaj et al., 2016). A previous study revealed that *Rhizobium* and *Bradyrhizobium* are major members of microbiomes in multiple compartments, including root endosphere, yet they cannot nodulate *Medicago* (Brown et al., 2020). The root endophyte microbiome is not an opportunistic subset of the rhizosphere microbiome but may be selected by complex processes and influenced by many factors, such as plant genotype and root exudates (Bacilio-Jiménez et al., 2003; Lundberg et al., 2012). The entry mode and function of Bradyrhizobiaceae enriched in the endosphere need to be further studied. The mutation of *noeI* did not affect the nodulation process in *B. diazoefficiens* USDA 110 under the sterile vermiculite condition (Liu et al., 2018). However, there was a significant reduce in nodulation efficiency of USDA 110 under the soil condition, since the ecological adaptability of USDA 110 was affected by complex microbial interactions and abiotic factors in soil. The decrease of nodulation efficiency of the mutant is reflected in the decrease of nodule number. Once the mutant infected the root through root hairs, after overcoming the environmental interactions, there was no difference in the relative proportion of mutant in unit mass of nodules compared to WT rhizobia. Thus, there was no significant difference in *Bradyrhizobium* abundance between WT and mutant-induced nodules.

The bacterial families significantly enriched in nodules inoculated with WT strain and in soil supplemented with the flavonoid mixture are presented in Figures 3C, 5B. These families included Burkholderiaceae, which contained some species able to form symbiosis with certain legumes from the *Papilionoideae* subfamily (Bontemps, 2010) and also some species that dominate soybean nodules (Ramirez et al., 2019), and species known as plant growth-promoting bacteria in non-legume plants (Touceda-Gonzalez, 2015). Our results are consistent with other studies wherein there was a depletion of Burkholderiales in the roots of *Lotus* when inoculated with symbiosis pathway gene mutants (Zgadzaj et al., 2016; Thiergart et al., 2019). In contrast, we found a significant depletion of Sphingobacteriaceae and Burkholderiaceae in the rhizosphere and rhizoplane of plants inoculated with the WT strain, which might be a consequence of potential niche replacement as a compensatory effect following the enrichment of Micromonosporaceae and Streptomycetaceae in these compartments.

### The Microbial Co-occurrence Networks Shaped by *noeI*

Network analysis, an approach to visualize and examine microbial abundance patterns, confirmed a gradient of decreased diversity observed from soil to root and nodule compartments. This pattern is also reflected in the topological features of the sub-networks. We noticed higher average degree, connectivity and number of clusters, and lower average path length and diameter for OTUs in the WT networks compared to the *noeI* mutant. This observation is possibly linked with the enhanced diversity seen in in root-associated compartments of the WT treatment. For instance, the higher average degree indicates that there are more potential bacterial connections in samples inoculated with the WT strain than those in the *noeI* mutant inoculated samples, average degree measures the number of direct co-occurrence links for an each OTU in the network (Greenblum et al., 2012). Our results are consistent with other work showing that rhizobia inoculation lead to an increase in soybean rhizobacterial network connections (Zhong et al., 2019). The increased modularity and number of clusters from bulk soil to nodule supports the conclusion that the nodule compartment is a highly selective niche (Zgadzaj et al., 2016). Co-occurrence networks also identified several microbial clusters, which were composed of Rhizobiaceae and Clostridiaceae_1 (Saito and Minamisawa, 2006). Taken together, the network analysis suggested that functional symbiosis structures a more tightly connected bacterial network.

### The Effect of Flavonoid Exudates and Edaphic Factors on Microbial Community Shift

As a group of plant secondary metabolites, flavonoids present various bioactivities like anti-oxidative and anti-microbial activities (Panche et al., 2016). In the symbiosis between legumes and rhizobia, flavonoids secreted by roots of legume functioned as molecular signals for rhizobial recognition and colonization to their specific hosts. Therefore, the composition and amounts of flavonoids might be a factor affecting both the symbiotic bacteria and the other rhizomicrobes. To that end, a comparative analysis of flavonoids secreted by soybean plants inoculated with wild-type and *noeI* mutant strains was conducted. Previously, the most frequently used method for extraction of flavonoids in root exudation was determined with plants cultured in hydroponic solutions (Dardanelli et al., 2008). This method might generate flavonoid patterns that differ from those in soil since the soil characters, like N-nutrient content, had a great influence on the secretion of flavonoids (Coronado et al., 1995). Meanwhile, the interactions of flavonoids with surface-reactive soil particles such as hydrophobic humic molecules (for hydrophobic flavonoids) and clay minerals (for hydrophilic flavonoids) can affect the structure and behavior of flavonoids, and lead to incomplete recovery of flavonoids from the soil when the analysis of flavonoid secretion is performed in plants cultured in the soil (Cesco et al., 2010). We addressed these limitations by using a hybrid collection method to make the detected flavonoid pattern more similar with *in situ* analysis (Williams et al., 2021), where soybean plants were grown in sterile vermiculite and transferred to a hydroponics containing same amount of inorganic nitrogen as that in the soil. In this analysis, we found daidzein as the most abundant flavonoid secreted by soybean variety C08, followed by coumestrol and genistein. This is consistent with the previous reports on root exudates under similar conditions (Ramongolalaina, 2019) and for most soybean cultivars (Cesco et al., 2010). Previous studies reported that the amounts of secreted flavonoids were increased by inoculation with compatible symbionts or by treatment with Nod factors and were reduced by inoculation with *nodC* mutant rhizobium (Bolaños-Vásquez and Werner, 1997; Cesari et al., 2019). Accordingly, we found a significantly increased exudation of most flavonoids when inoculating the WT strain, which was not observed for the *noeI* mutant and is most likely due to the defective symbiosis. This difference might be a reason why fewer nodules were formed and a different microbiome was detected for the plants inoculated with *noeI* mutant in comparison with those of WT inoculated plants.

Root exudates present a major organic carbon resource for soil microorganisms and drive the assembly of plant rhizosphere microbial communities. Specific compounds in exudates are thought to promote or suppress specific soil microbial members, leading to the formation of distinctive root-associated microbiomes (Ramongolalaina, 2019). We found that the exogenous supplementation of flavonoids affected soil microbiome diversity compared to the control-treated soil; this is consistent with other studies that have revealed the variation in soybean microbial communities in relation to the application of daidzein and genistein (Ramongolalaina, 2019; Okutani et al., 2020). We found a significant decrease of flavonoid secretion in soybean roots inoculated with *noeI* mutant strain and a microbial community shift in soil with exogenous supplementation of flavonoids. However, there was no direct evidence to connect the variation of root-associated microbial community mediated by *noeI* mutant to flavonoid secretion shifts. Therefore, flavonoids may act as just one of the influencing factors related to microbial community shift under different rhizobial treatments, and other important factors to be verified in further study. Redundancy analysis identified that environmental factors represented by daidzein and soil exchangeable Mg^2+^ were significantly associated with the rhizosphere microbial shift. It is not surprising that soil physiochemical properties impact the soil microbiome (Xiong et al., 2012; Ma et al., 2017). The root exudates interact with edaphic properties to form a distinctive environment from the surrounding soil that promotes microbial growth and proliferation, shaping a more diverse community (Bulgarelli et al., 2013; Lareen et al., 2016). This may explain why not only root exudates but also soil physiochemical properties have effects on soil microbial communities. These results were only based on the analysis of limited influencing factors we measured, and there are still many unknown factors likely affecting microbial communities in this process to be studied.

## Conclusion

**Figure 6:**
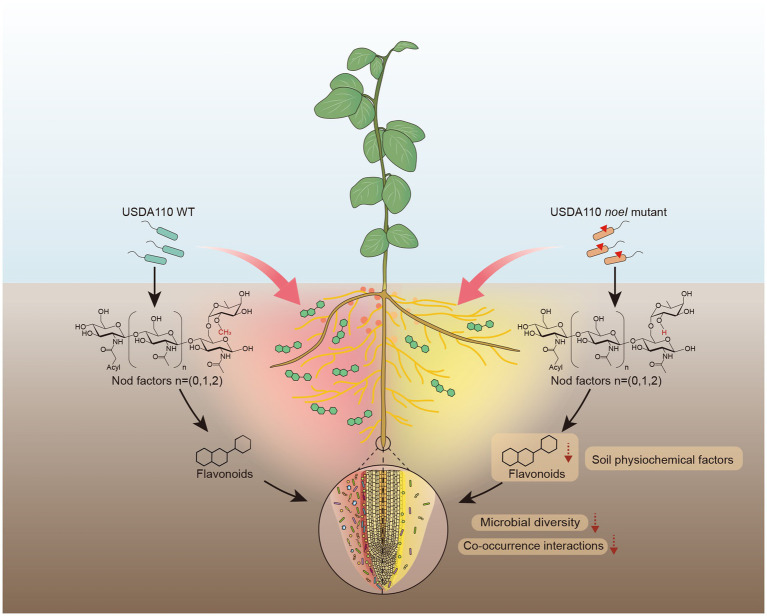
Proposed mechanisms underlying how rhizobium with mutated nodulation gene *noeI* affects soybean root-associated microbiomes.

In summary, our data point to the following model (Figure 6): rhizobium with an intact *noeI* gene (WT) promotes a higher symbiotic capacity with soybean, such as more nodules and higher nitrogenase activity compared to the mutant strain. Further, the symbiotic capacity shapes the root-associated microbial communities, which include the significantly reduced microbial diversity and co-occurrence interactions mediated by *noeI* mutation. Flavonoid exudation and soil physiochemical factors were found to be associated with the rhizosphere microbial community shifts under the different rhizobial treatments. Understanding the consequences of the interplay (legumes, rhizobia, and root-associated microbiomes) for plant performance and the evolutionary dynamics of symbiosis are exciting prospects of this work.

## Data Availability Statement

The amplicon sequencing datasets were submitted to Genome Sequence Archive (GSA) and are accessible under the project accession number PRJCA002971.

## Author Contributions

JX and BM conceived and supervised the study. JX, BM, YL, WC, and EW designed the experiment. YL collected samples and extracted DNA. YL, BM, and KZ analyzed the data. YL and ZL performed the root exudate collection and UPLC-MS/MS analysis. YL and SY performed visualization of the data. YL wrote the first draft of the manuscript. BM, WC, ES, KS, LH, ME, EW, YZ, and JX revised the manuscript. All authors have read and approved the final version of the manuscript.

## Funding

This study was funded by the National Natural Science Foundation of China (41991334, 41721001), the 111 Project (B17039), and the Fundamental Research Funds for the Central Universities (2020XZZX002-18).

## Conflict of Interest

The authors declare that the research was conducted in the absence of any commercial or financial relationships that could be construed as a potential conflict of interest.

## Publisher’s Note

All claims expressed in this article are solely those of the authors and do not necessarily represent those of their affiliated organizations, or those of the publisher, the editors and the reviewers. Any product that may be evaluated in this article, or claim that may be made by its manufacturer, is not guaranteed or endorsed by the publisher.
