# Supplementary material for: Rhizobium Symbiotic Capacity Shapes Root-Associated Microbiomes in Soybean
Source: Front Microbiol. 2021 Dec 2;12:709012. doi: 10.3389/fmicb.2021.709012 (PMC8678110; doi:10.3389/fmicb.2021.709012)
Supplement: Supplementary file 1 [file Data_Sheet_1.pdf]

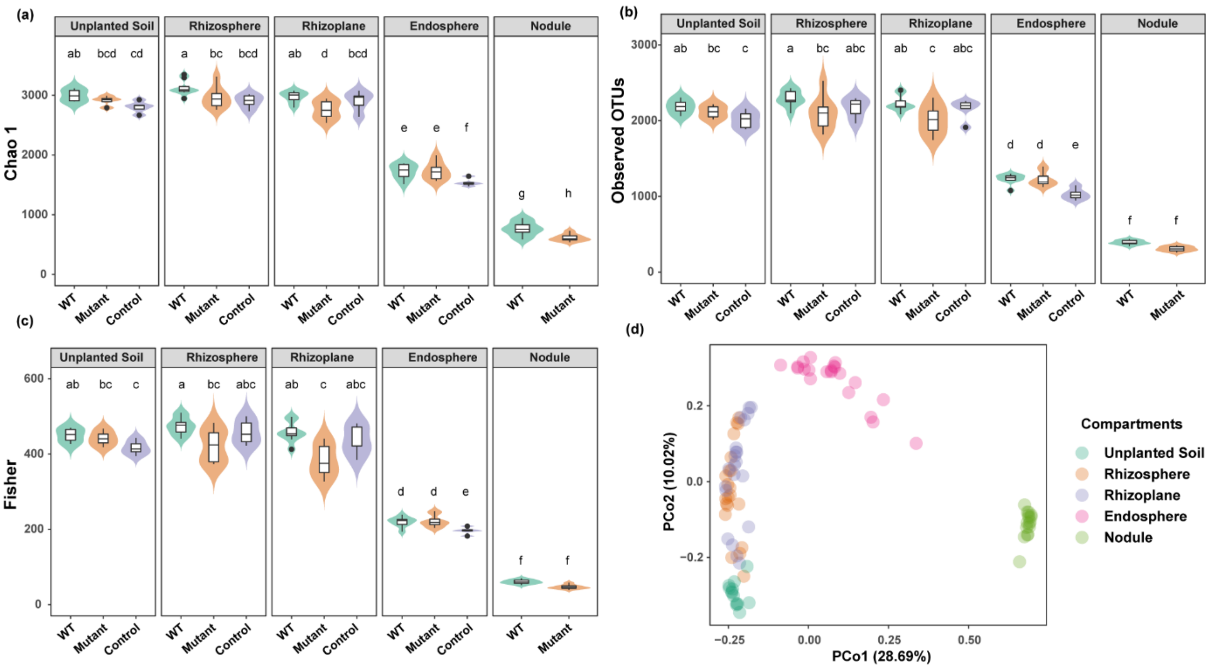

Fig. S1. α-diversity and β-diversity among different rhizobial treatments in unplanted soil, rhizosphere, rhizoplane, endosphere and nodule. α-diversity among different rhizobial treatments in unplanted soil, rhizosphere, rhizoplane, endosphere and nodule as determined using chao1 (**a**), observed OTUs (**b**) and Fisher (**c**). Treatments are wild type (WT), *noeI* mutant (Mutant) and uninoculated (Control). Different letters represent significant differences among treatments (Dunn's multiple-comparison test, $p < 0.05$). (**d**) β-diversity principal coordinate analysis (PCoA; weighted UniFrac distances) of unplanted soil, rhizosphere, rhizoplane, endosphere and nodule communities.

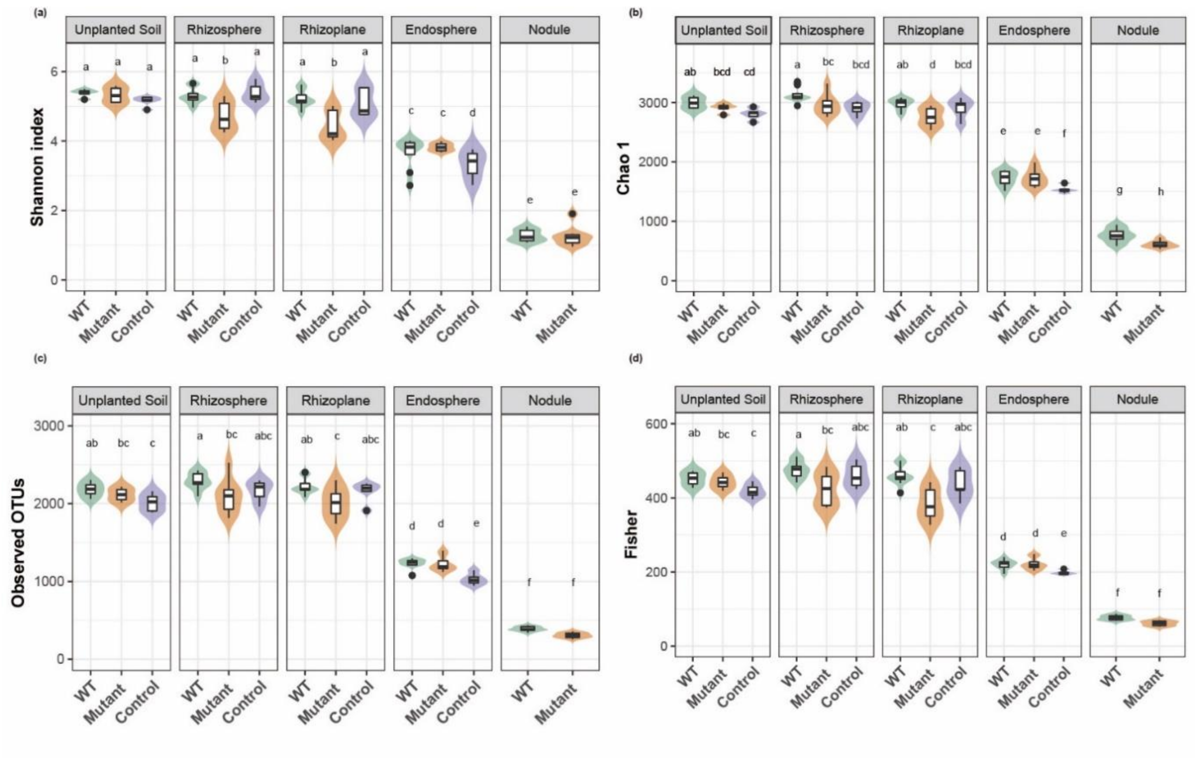

Fig. S2. α-diversity (Shannon, Chao1, Observed OTUs and Fisher indices) among different rhizobial treatments in the unplanted soil, rhizosphere, rhizoplane, endosphere, and nodule compartments in the absence of the 16S rRNA sequences of *B. diazoefficiens* USDA 110 (OTU_77). Treatments are wild-type USDA 110 (WT), *noeI* mutant (Mutant), and not inoculated with rhizobia (Control). Different letters indicate significant differences among treatments (Dunn's multiple-comparison test; $p < 0.05$).

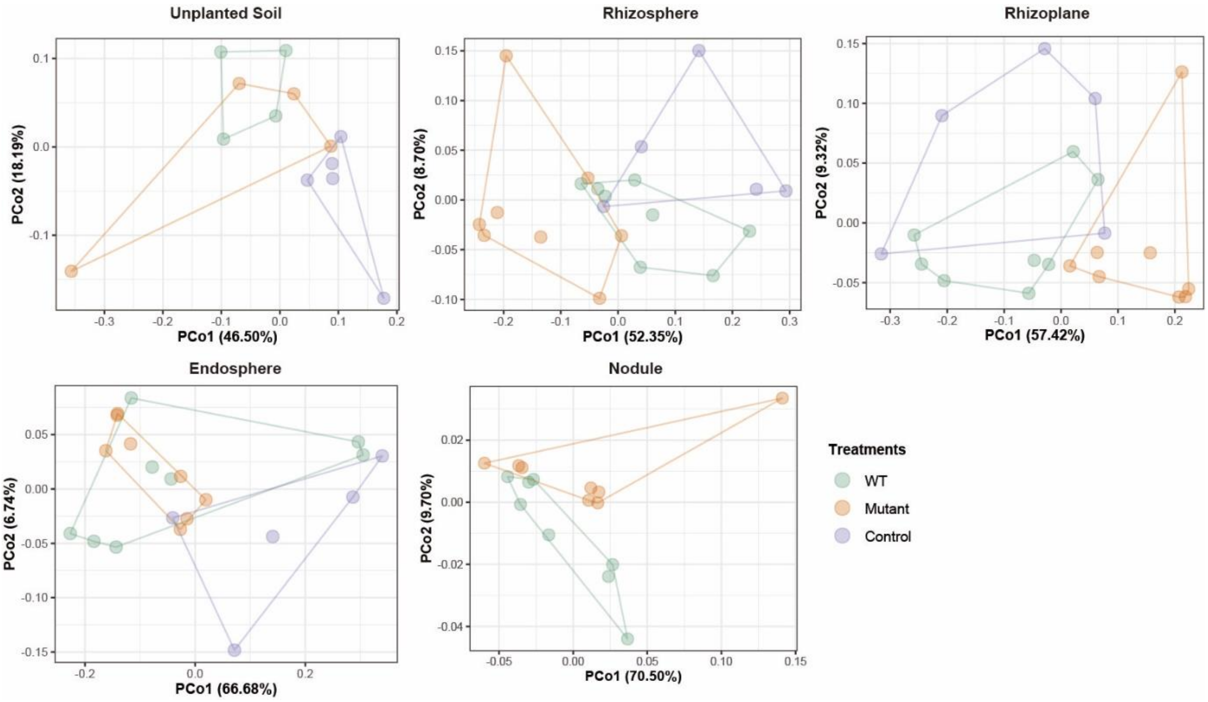

Fig. S3. **Principal coordinate analysis (PCoA; weighted UniFrac distances) of unplanted soil, rhizosphere, rhizoplane, endosphere and nodule communities of soybean among different rhizobial treatments in the absence of the 16S rRNA sequences of *B. diazoefficiens* USDA 110 (OTU_77).** Treatments are wild-type USDA 110 (WT), *noeI* mutant (Mutant), and not inoculated with rhizobia (Control).

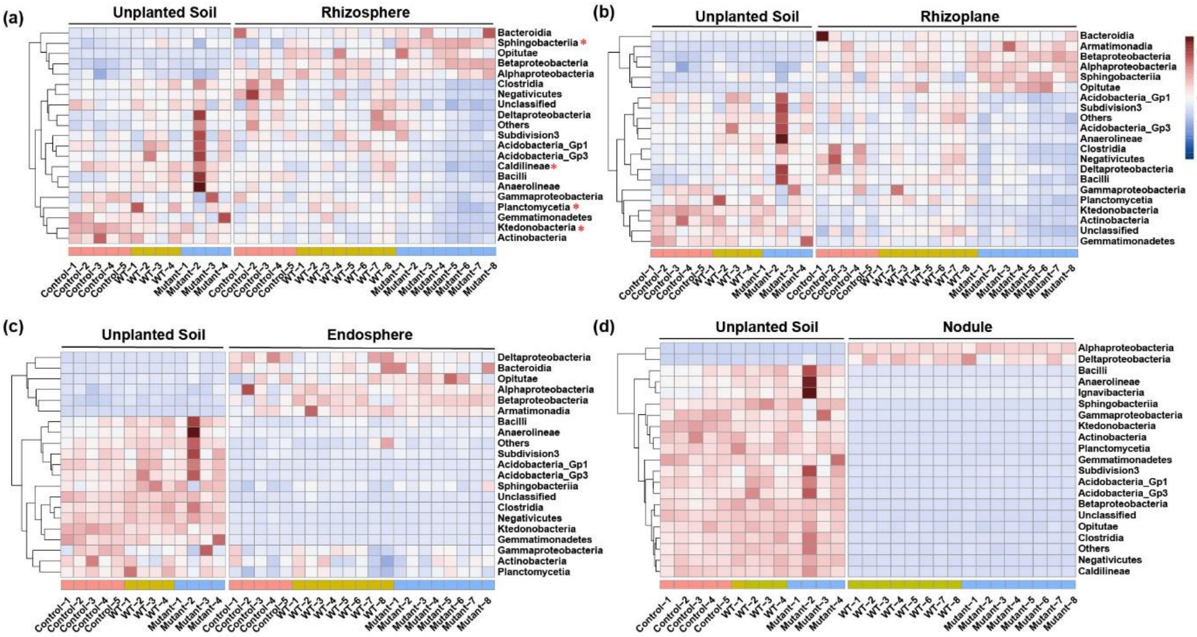

Fig. S4. Microbial community composition in rhizosphere, rhizoplane, endosphere and nodule. Microbial community composition (class) in wild type (WT), *noeI* mutant (Mutant) and uninoculated (Control) samples using the unplanted soil (subset) compared with rhizosphere (**a**), rhizoplane (**b**), endosphere (**c**) and nodule (**d**). Color gradation indicates relative abundance, * represents the classes significantly differed between WT and mutant treatment (Duncan multiple-comparison test, $p < 0.05$).

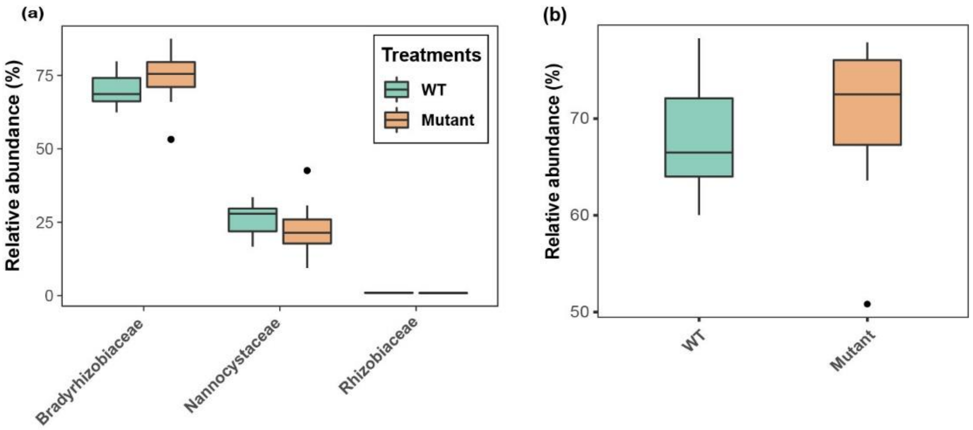

Fig. S5. The relative abundance of the dominant taxa in the nodule samples. (**a**) The relative abundance of the top abundant taxa (at the family level) in the nodule samples treated with WT and mutant rhizobia (n = 8). (**b**) The relative abundance of *B. diazoefficiens* USDA 110 in nodule samples treated with WT and mutant rhizobia (n = 8).

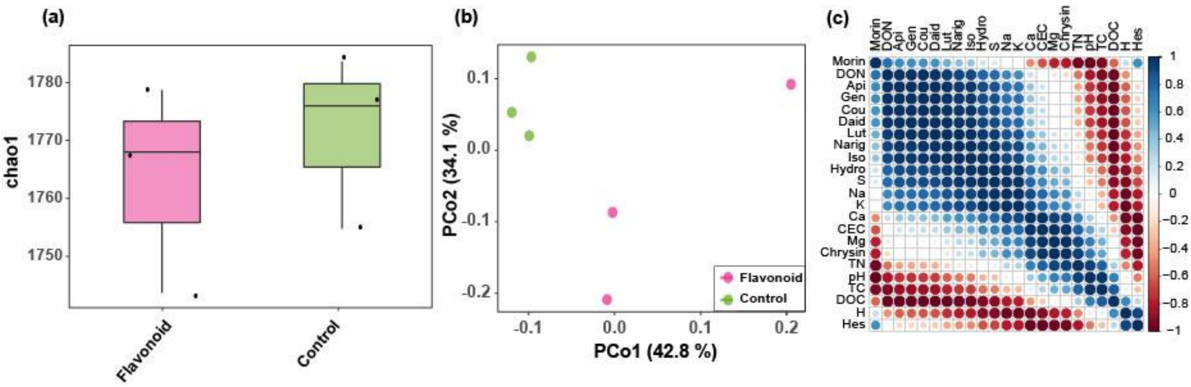

Fig. S6. α-diversity and β-diversity in soil inoculated with a mixture of flavonoids and the uninoculated control. (**a**) α-diversity (Chao1) in soil inoculated with a mixture of flavonoids (Flavonoid) and the uninoculated control (Control). (**b**) PCoA (weighted UniFrac distances) of soil treated with flavonoids or water (control). (**c**) Correlation analysis of the environmental factors, including carbon and nitrogen related indexes (TC, TN, DOC, DON), and alkalinity related parameters (pH, exchangeable base cations, cation exchange capacity) and root exudates: 7, 4′-dihydroxyflavone (Hydro); genistein (Gen); daidzein (Daid); luteolin (Lut); isoliquiritigenin (Iso); apigenin (Api); coumestrol (Cou); naringenin (Narig); hesperetin (Hes); morin and chrysin.

Table S1. Mass spectrometry parameters and ion patterns of tested compounds

Compound	Parent (m/z)	Daughter1 (m/z)	Daughter2 (m/z)	Dwell (s)	Cone voltage (V)	Collision energy1 (eV)	Collision energy2 (eV)
7, 4′-dihydroxyflavone	160.9	104.9	132.9	0.025	14	22	18
Chrysin	253.0	142.9	208.9	0.025	16	26	22
Daidzein	253.0	208.1	223.9	0.025	16	30	26
Isoliquiritigenin	255.1	119.0	134.9	0.025	28	22	16
Coumestrol	266.9	238.9	266.8	0.025	12	24	22
Apigenin	285.0	120.9	134.9	0.025	8	26	22
Genistein	269.0	132.9	158.9	0.025	26	30	30
Naringenin	271.0	118.9	150.9	0.025	32	24	18
Luteolin	285.0	132.9	150.9	0.025	36	34	26
Morin	301.0	124.9	150.9	0.025	8	18	18
Hesperetin	301.1	163.9	286.0	0.025	16	24	16
Genistein-D2	271.0	134.9		0.025	8	32	

Table S2. The effects of environmental variables on the microbial community assembly revealed by permutational multivariate analysis of variance (PERMANOVA).

	SS[c]	F. Model	Variation (%)[d]	*p* value[e]
Treatments	2.206	6.596	12.91	0.001***
Compartments	6.671	13.928	39.04	0.001***
Treatments × Compartments[a]	3.134	6.740	18.34	0.001***
Rhizosphere -Treatments[b]	0.312	5.969	39.87	0.001***
Rhizoplane -Treatments	0.323	5.263	36.90	0.003**
Endosphere -Treatments	0.226	3.101	25.63	0.03*
Nodule -Treatments	0.007	0.817	5.51	0.398
Unplanted Soil -Treatments	0.114	1.662	24.95	0.078

[a] "×" indicates interactions between these environmental variables.
[b] "-" represents the effect of treatments in each compartment on the microbial community assembly.
[c] SS, sums of squares.
[d] Variation was based on weighted UniFrac distances.
[e] *p* value based on PERMANOVA (999 permutations).
* $p < 0.05$
** $p < 0.01$
*** $p < 0.001$.

Table S3. Topological features of subnetworks where 'WT' represents the USDA 110 wild type treatment and 'Mutant' represents the *noeI* mutant treatment.

	Bulk Soil	Rhizosphere		Rhizoplane		Endosphere		Nodule	
**Topological features**		**WT**	**Mutant**	**WT**	**Mutant**	**WT**	**Mutant**	**WT**	**Mutant**
Modularity	0.455	0.508	0.570	0.511	0.571	0.528	0.540	0.632	0.642
Number of vertices	302	282	279	283	279	276	266	212	203
Number of edges	3426	2490	2259	2501	2263	2298	1990	1223	1096
Connectance	0.075	0.063	0.058	0.063	0.058	0.061	0.056	0.055	0.053
Average degree	22.689	17.660	16.194	17.675	16.222	16.652	14.962	11.538	10.798
Average path length	3.369	3.862	4.663	3.861	4.663	3.867	4.680	4.207	5.590
Diameter	9.000	26.681	35.577	26.681	35.577	26.681	35.577	26.897	39.990
Clustering coefficient	0.513	0.551	0.572	0.553	0.572	0.519	0.537	0.527	0.570
Number of clusters	2	4	3	4	3	4	3	4	3
Degree centralization	0.077	0.065	0.078	0.065	0.078	0.070	0.072	0.069	0.051
Closeness centralization	0.009	0.011	0.009	0.010	0.009	0.010	0.012	0.013	0.011
Betweenness centralization	0.111	0.150	0.151	0.149	0.151	0.148	0.156	0.160	0.224
